# Statewide Cancer Drug Repository Reducing Waste in the Setting of Shortage Crisis

**DOI:** 10.1093/oncolo/oyae040

**Published:** 2024-03-14

**Authors:** Emily R Mackler, Siobhan Norman, Farah Jalloul

**Affiliations:** YesRx, Ann Arbor, MI, USA; Michigan Oncology Quality Consortium (MOQC) and Michigan Institute for Care Management and Transformation (MICMT), Ann Arbor, MI, USA; University of Michigan College of Pharmacy, Ann Arbor, MI, USA; YesRx, Ann Arbor, MI, USA; YesRx, Ann Arbor, MI, USA; Michigan Pharmacists Association, Lansing, MI, USA

**Keywords:** cancer drug repository, medication waste, financial toxicity in cancer, equitable cancer care

## Abstract

Disparities in cancer treatment, including access to medications, continue to exist. Rising drug prices and cancer drug shortages are 2 causes of inequitable access to treatment. This article introduces pilot outcomes for a solution to improve access to medications while also decreasing medication waste. Cancer drug repositories are an innovative patient-centered model where donations of unused cancer medications from patients are repurposed and provided to patients who are most vulnerable and disproportionately harmed by financial toxicity. This model demonstrates efficiency and sustainability that complements integrated care and provides an approach to increase medication access and decrease medication waste.

## Teaser

Rising drug prices and cancer drug shortages are 2 causes of inequitable access to cancer treatment. This article introduces pilot outcomes for a solution to improve access to medications while also decreasing medication waste.

**Implications for Practice**

Cancer drug shortages and high-cost cancer medications can limit the ability of patients with cancer to receive the medications they need. Yet, astonishing cancer medication waste is occurring, with millions of dollars of cancer medications discarded each year. To address this, we describe an innovative model of cancer drug repositories where patients can donate their unused cancer medications to people who are most vulnerable and impacted by financial toxicity that prevents them from receiving the life-saving medications they need. This patient-centered model reaches across the State of Michigan and is structured to facilitate other patient access issues, including those that arise during cancer drug shortages. In practice, this model increases equitable patient access to medications while decreasing harmful waste.

## Introduction

The complexity and implications of cancer drug shortages have been well described in this issue and other recent publications.^1^ Drug shortages lead to multiple consequences, including delays, changes, or cancelations of treatments; increased health care resource needs; drug safety concerns; and increased costs to the health care system.^1,2^ In the cancer patient population, the most severe consequence of national shortages is patients not receiving the medications they need to treat their cancer, leading to a plethora of negative outcomes. Data demonstrate that the risk of shortage is particularly high for generic drugs, where the production processes remain complex and costly while the margins are much lower than branded medications.^2,3^ On the other hand, patients are regularly at risk of not receiving the brand drugs they need due to the high cost and resulting financial toxicity.^4,5^ In the midst of both these scenarios, astonishing waste is occurring, with millions of dollars of cancer medications being discarded annually.^6^

People with cancer are at particularly high risk for experiencing financial toxicity, with nearly half (49%) of adults with cancer reporting material or psychologic financial burdens.^7^ A major contributor is oncology drug therapy, where the median annual cost for one oncology drug course is $196 000 (IQR, $170 000-$277 000).^8^ Spending on cancer medications is expected to increase to a projected amount of $375 billion globally by 2027, up from $196 billion in 2022 (almost double in 5 years).^9^ Financial burden continues to increase due to changes in treatment coverage, often requiring greater cost sharing by the patient.^10-12^ This is especially challenging for patients receiving treatment with oral anticancer agents (OAAs), as they are typically covered under the prescription drug benefit rather than the medical benefit of most insurance plans.^13,14^ For Medicare Part D specifically, patients receiving OAA treatment quickly reach their coverage gap (donut hole) and catastrophic coverage based on out-of-pocket spending, which in 2023 is at $7400.^15^ This is just over half the 2023 national poverty level of $14 580 per individual.^16^ This is of particular importance given the average age of the cancer population, many of whom fall within Medicare coverage. That first initial coverage of the plan has the patient pay only copays for their medications but once they reach the designated limit of medication coverage, they enter what has been coined as the “donut hole” where they pay a percentage of the drug cost until they are eligible to begin receiving the catastrophic coverage. Given the cost of OAAs, it is common for patients to experience this gap in coverage. Of Medicare beneficiaries receiving OAA therapy and not eligible for low-income subsidies in 2016, 60% reached the catastrophic phase of coverage with most patients reaching it the same month they initiate their OAA therapy.^17^

Not surprisingly, financial toxicity further perpetuates cancer inequities.^18^ Individuals from rural communities experience disparities in cancer incidence and outcomes, including survival and also experience a higher degree of financial toxicity than their urban counterparts.^19^ In a recent study of patients taking OAAs specifically, those living in rural or suburban areas (rather than urban) experienced worse financial toxicity and also experienced increased symptom burden, including fatigue, emotional distress, insomnia, and lack of appetite.^20^

To better understand the implications of drug cost on the ability of patients with cancer to take OAAs as prescribed, prescription abandonment rates have been studied. First-fill abandonment rates are determined by examining cases where the OAA is initially prescribed, submitted to the pharmacy, and then has had the insurance claim reversed after adjudication with no patient follow-up or receipt of the prescribed medication.^21^ OAA abandonment rates have been reported at a frequency of 10%-20%.^21-23^ Higher percentages of patient cost sharing have been shown to increase medication abandonment rates while decreasing initiation and persistence of therapies.^24^ Financial toxicity is a critical barrier to a patient’s ability to receive the best care available for their cancer—at every spectrum of their journey—upfront initiation, subsequent therapies, and palliative care.

Patients and clinicians face difficulty in receiving the cancer medications they need due to drug shortages or financial toxicity, at the same time, medication waste is on the rise. A report by ProPublica in 2017 details how extensive medication waste is likely to be. For example, they report that Colorado long-term-care facilities throw away over 17 tons of potentially reusable drugs each year (worth approximately $10 million) and that the Environmental Protection Agency estimated 740 tons of drug waste by nursing homes in 2015.^25^ This does not take into consideration waste from patient homes or other health care facilities. Although data are sparse for cancer treatments specifically, we know a significant amount of drug waste exists due to OAA regimen changes required in response to cancer response and/or treatment side effects. A recent study by Lam et al^26^ estimated an average of $4290 (SD, $5720) of drug waste per patient taking an OAA. The American Society of Clinical Oncology (ASCO) estimates over $3 billion of cancer medication waste annually.^6^

**Figure 1.**
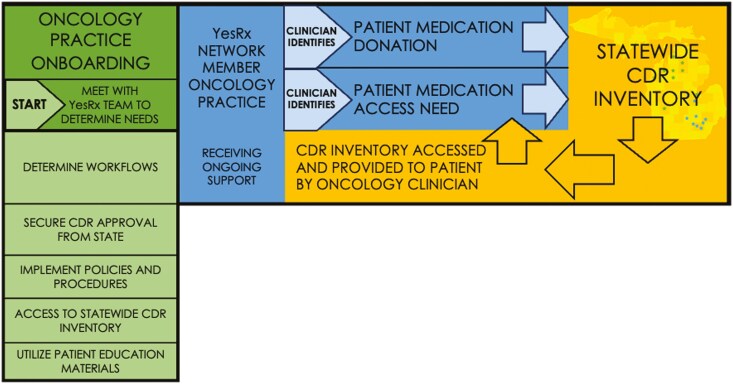
YesRx statewide CDR network process.

The scenario described above provides the opportunity to improve patient access to care while decreasing waste. Both objectives align with the United Nations Sustainable Development Goals, in which there is a focus on improved health (goal 3), reduced inequalities (goal 10), and responsible consumption and production (goal 12).^27^ This juxtaposition of medication need and simultaneous waste creates an opportunity to soften both implications and decrease inequities in medication access via cancer drug repositories (CDRs; Fig. 1).

## Innovation: CDRs

The overall financial burden of cancer and its distressing consequences are now generally acknowledged as a priority concern faced by patients and oncology providers. Yet, an effective response to this problem remains lacking.^7^ Cancer drug repositories are one innovative solution to explore. CDRs have gained interest as a means to alleviate some of the challenges associated with drug affordability, access, and waste.^6^ They are born out of the growing pervasiveness of financial toxicity. CDRs are specialized facilities that collect, store, and redistribute unused cancer medications and other supportive/cancer-related medications that qualify for repurposing. These facilities play a vital role in addressing the issue of drug waste in cancer by allowing for the repurposing of qualifying OAAs. Through structured and stringent quality control measures, CDRs monitor and control the safe and ethical transfer of cancer drugs from patients who no longer need the medication to another who is in need. By doing so, CDRs reduce oncology drug waste, relieve financial distress, and enable medication access for patients while promoting sustainability within health care.^6,28,29^

Several states have established CDR program legislation, each with its own set of regulations and limitations (Table 1).^30^ Each state differs in its approach and restrictions imposed. For example, all states mention that an eligible patient must be indigent; however, states like Michigan will also allow for dispensing to any resident with a cancer diagnosis if the drug cannot be dispensed to those uninsured or underinsured before the drug expires.^31^ Although legislation is determined at the state level, program implementation varies from site-specific to statewide. An example of a site-specific program is the recently implemented oral oncology drug repository program at the James Cancer Hospital at The Ohio State University Wexner Medical Center (OSUWMC) where a pilot was launched in 2020. OSUWMC’s program evaluated multiple donations and redispensing scenarios during their pilot with the plan to expand with additional resources.^29^ Finally, some states may not have separate CDR programs but actively collect and redistribute cancer medications as part of their general drug repository program. Notably, Iowa’s statewide program, SafeNetRx, established in 2007, and Minnesota’s statewide program, RoundTableRx, established in 2017, are longstanding drug repositories that include oncology medications among other chronic care and specialty medications.^32,33^ Since SafeNetRx’s founding, they have provided prescriptions to 134 000 patients in need, at no cost.^32^

**Table 1.** States with specific cancer drug repository program in addition to general repository program legislation.^27^

US States
California
Michigan
Nebraska
Pennsylvania
Wisconsin
Florida
Minnesota
Nevada
Utah
Kentucky
Montana
Ohio
Washington

In Michigan, the legislation related to CDRs has been in effect since 2006.^31^ In November 2021, the first CDR site was established in the State within a community oncology practice by an oncology clinical pharmacist who was embedded within the practice as part of the program, POEM (Pharmacists Optimizing Oncology Excellence in Michigan).^31,34^ Soon after, 2 additional community oncology sites, also in the POEM program, established their own CDRs within their pharmacy departments. The interprofessional integration of clinical pharmacists via POEM at community-based sites across the State allows pharmacists to join the clinical care team, providing direct patient care and improving outcomes for oncology patients including those receiving treatment with OAAs and patients from rural communities.^35-37^ In fact, the majority (90%) of the pharmacists engaged in the POEM program chose their primary clinical focus to be the education and collaborative management of patients receiving OAAs. POEM was launched in 2020, via the partnership between the Michigan Oncology Quality Consortium (MOQC) and the Michigan Institute for Care Management and Transformation (MICMT), quality improvement programs engaged with oncology practices and physician organizations respectfully across the State.^38,39^ The development and pilot testing of CDRs was a fortuitous outcome of having embedded clinical oncology pharmacists in the practices, resulting in improved access to medications for underinsured and noninsured patients. Collectively the CDRs received over 150 medication donations valued at $3 million in a 18-month period. During that time, the CDRs provided medications at no cost to 30 patients in need. These unprecedented pilot successes revealed clear needs for sustainability that could not be met without significant support. Increased resources in personnel and space were needed at each site to maintain the CDR programs. Additionally, all sites expressed growing concern related to the possibility of drugs expiring out while in the repository inventory. The implementation barriers just mentioned may have also been contributors to the delayed development of CDRs in the State, resulting in the long gap from legislation to the establishment of the first CDR sites. In addition, our group hypothesizes that the nature of the POEM program providing critical clinical pharmacy resources at community sites allowed for the identification of patient need, understanding of pharmacy operational function, and clinical and operational expertise to implement the CDR programming. YesRx, a 501(c)3, charitable medication access service organization formed in June 2023, provides essential support to streamline CDR program operations, connects cancer clinicians across a statewide network of CDR programs, and develops a perpetual shared CDR inventory of medications available for clinicians to access for patients—at no cost. (Fig. 1)^40^ The result is the YesRx Network, a consortium of CDR sites in Michigan expanding patient access to OAAs and supportive medications through collaboration. YesRx seeks to remove barriers to medication access for vulnerable and underserved people and communities by optimizing CDR use and minimizing drug waste. To our knowledge, this is the first statewide repository program for cancer drugs, and specifically focused on building partnerships with oncology clinics and practices across the state to ensure that practices with the least resources can receive support that enables them to access this program for their patients.

Progress in the first 6 months of YesRx operations have highlighted the needs a statewide CDR program meets. Several milestones were achieved in this time including critical sponsorship. Support for the first year of operations was provided in funds from private individuals and Blue Cross Blue Shield of Michigan through the Value Partnerships program and a pharmacy location for central CDR storage and operations provided by Trinity Health System in Ypsilanti, Michigan. The YesRx Network grew from the initial 3 practices (9 sites in total) to 18 total participating sites, expanding the reach across the State. YesRx received over 125 patient medication donations (valued at $2.4 million AWP), and dispensed medications to 106 patients at no cost (valued at $1.3 million AWP) during the 6-month period.

**Figure 2.**
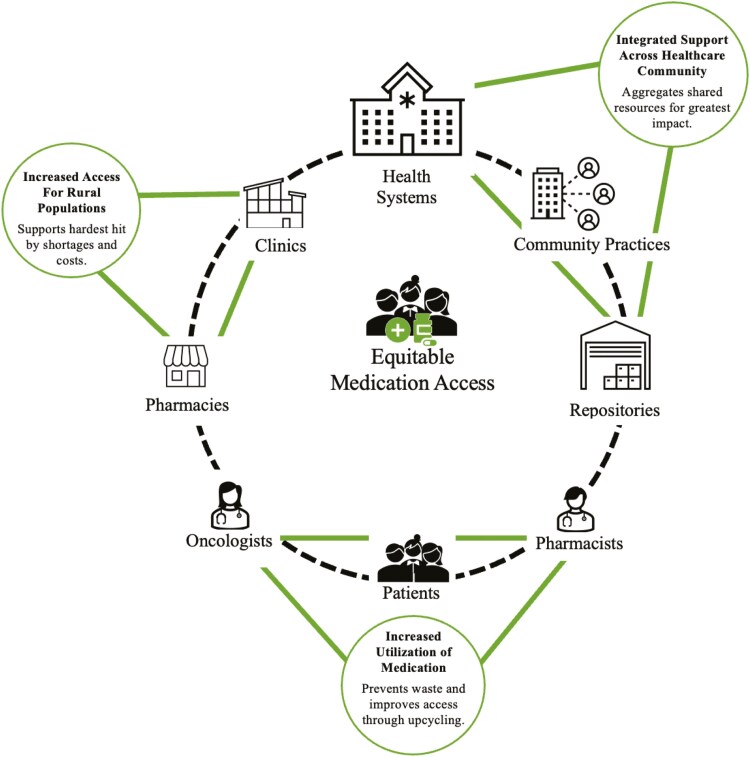
Aspects of a CDR network as an ecosystem approach for equitable medication access and a conceptual model for drug shortage mitigation.

Finally, this CDR model (Fig. 2) unites health care clinicians who share a vision of increasing medication access to patients with cancer. The network of CDRs developed an infrastructure where clinicians communicate patient needs and are able to collaborate in a shared vision of providing donated medications to vulnerable patients. This same infrastructure can be leveraged for alerting needs during drug shortages. Thus, the collective inventory, expertise, processes, and communication tools created by the CDR network can be used to further decrease inequities in cancer care.

## Conclusion

Challenges to equitable access to cancer treatment continue with the increasing incidence of drug shortages and rising medication costs. Solutions to both problems will require multifaceted approaches, including legislative and large system changes. In the meantime, reducing the rising waste of cancer medications via redispensing those medications to offset the financial toxicity experienced by patients is a novel intervention that has already returned promising outcomes.

We recognize limitations of this innovative model, including the variability of program feasibility due to differing state laws. Preferably, a central, regional, or national consistent model would be ideal to reduce resource requirements. However, most legislation allows collection and redispensing to occur at the state level, and states have different guidance for patient and site eligibility. Another limitation of the collection is related to how oral cancer medications are originally dispensed to patients. Ideally, medications would be packaged in unit dose systems to allow for the safest, most inclusive collection of unused medications. For example, in Michigan, the law does not allow for the redistribution of medications that have already been opened due to the inability to ensure product safety. How medication waste could be mitigated by manufacturers needs to be further explored related to packaging and product availability.^41^ Strategies to minimize waste with intravenous chemotherapy have already been discussed related to provision of medications in multidose rather than single-dose vials and additional availability addressing most common doses administered.^42^ Similarly, analysis should occur of how best to provide oral anticancer medications to patients where dose titrations, standard dosing, and potential for repurposing are all considered in packaging.

Limitations also exist related to the equitable distribution of redispensed medications. Many factors can impact this. In some scenarios, the practices that have the resources (personnel, space, and finances) to support a CDR may also have other resources that are of great benefit to patients needing access to care including cancer financial navigators, social workers, and pharmacy services. Thus, patients in the greatest need may be receiving care at sites with less extensive resources. This was one of the first barriers we targeted to remove by developing the statewide network—the ability to support practices to enable access to CDR medications for their patients even if they are not as resourced as some of their larger counterparts. Practice variation in the identification of patients who would be eligible for CDR medication is another possible limitation. Our Network holds biweekly meetings and follows agreed-upon practices to minimize inequities that could arise with variations in patient identification. An example of this is our Network approach to fill 1 month or less of medication at a time to allow for gaps in coverage to be met via a first come, first serve basis. This intentional approach ensures that the financial support needed for long-term OAA treatment is occurring within the oncology practice, as the Network inventory does not currently have enough donations to cover patients for a full course of therapy. Finally, our model is in its infancy. Although, we hypothesize that the benefits of the Network infrastructure will allow for greater engagement and patient access across the State, ongoing outcome evaluation and sustainable funding sources will be needed to continue and improve upon the model and the support it provides.

We hypothesize these limitations also offer strength to propel our work forward and drive solutions that will improve medication access to patients while also decreasing waste. The success of our model will facilitate fluid communication and shared goals for positive patient outcomes. The benefits are clear from the pilot outcomes demonstrated above by the sites that launched the first CDRs in Michigan and from our first 6 months as an organized statewide network. The collaboration of these sites and the interprofessional leadership they have displayed has been the impetus for us to expand across the State. We believe this statewide CDR network model can be used in other times of needs to help efficient and effective resources reach our patients across the State—namely during cancer drug shortages.

## Data Availability

The data underlying this article will be shared on reasonable request to the corresponding author.
